# Male morphological traits are heritable but do not predict reproductive success in a sexually-dimorphic primate

**DOI:** 10.1038/s41598-019-52633-4

**Published:** 2019-12-24

**Authors:** Clare M. Kimock, Constance Dubuc, Lauren J. N. Brent, James P. Higham

**Affiliations:** 1 Center for the Study of Human Origins, Department of Anthropology, New York University, New York, NY USA; 2 Center for Research in Animal Behaviour, University of Exeter, Exeter, UK

**Keywords:** Ecology, Evolution

## Abstract

Sexual selection favours traits that increase reproductive success via increased competitive ability, attractiveness, or both. Male rhesus macaque (*Macaca mulatta*) morphological traits are likely to reflect the effects of multiple sexual selection pressures. Here, we use a quantitative genetic approach to investigate the production and maintenance of variation in male rhesus macaque morphometric traits which may be subject to sexual selection. We collected measurements of body size, canine length, and fat, from 125 male and 21 female free-ranging rhesus macaques on Cayo Santiago. We also collected testis volumes from males. We used a genetic pedigree to calculate trait heritability, to investigate potential trait trade-offs, and to estimate selection gradients. We found that variation in most male morphometric traits was heritable, but found no evidence of trait trade-offs nor that traits predicted reproductive success. Our results suggest that male rhesus macaque morphometric traits are either not under selection, or are under mechanisms of sexual selection that we could not test (e.g. balancing selection). In species subject to complex interacting mechanisms of selection, measures of body size, weaponry, and testis volume may not increase reproductive success via easily-testable mechanisms such as linear directional selection.

## Introduction

Sexual selection favours traits that increase reproductive success because they confer advantages in mating competition (intrasexual selection), mate choice (intersexual selection), or both^1,2^. Mating competition can take several forms, including direct contest competition (physical fights) and indirect competition^2^, whereby males do not compete through physical fights, but rather through mechanisms such as sperm competition (in which ejaculates compete within the female reproductive tract)^3^ and endurance rivalry (whereby males compete in endurance, by investing in mating effort over long periods of time)^2^. Where direct male-male mating competition (intrasexual competition) for access to fertile females is high, sexual selection promotes the evolution of traits such as large body size and weaponry (horns, antlers, or large canine teeth)^4,5^. Sperm competition selects for large testis volumes relative to body size and high levels of sperm production^3,6,7^. Where males compete through endurance rivalry, they invest in maintaining body condition^2^. Strong intersexual selection typically leads to the evolution of ornaments, like the bright coloration exhibited by many bird species^2^. These mechanisms do not act in isolation, however. An increasing number of studies have shown that intra- and intersexual selection may act on the same traits in a reinforcing or opposing manner (see Hunt *et al*.^8^). Furthermore, there is evidence for trade-offs between traits related to pre-copulatory selection (e.g., ornaments and weaponry) and post-copulatory selection (testis volume and ejuaculates)^9–12^.

The evolution of exaggerated male traits such as ornaments, large body size, weaponry, and large testis volumes, has been linked to sexual selection pressures across many species. Ornaments have been shown to impact male reproductive success (e.g., tail length and color in European barn swallow *Hirundo rustica*^13^, coloration in house finches *Carpodacus mexicanus*^14^, coloration in guppies *Poecilia reticulata*^15^). Weaponry and body size predict reproductive success in some (e.g., field crickets *Gryllus bimaculatus*^16^, American rubyspots *Haeterina americana*^17^, minnows *Phoxinus phoxinus*^18^, kangaroos *Macropus giganteus*^19^, red deer *Cervus elaphus*^20^, Soay sheep *Ovis aries*^21^, mandrills *Mandrillus sphinx*^22^), but not all (e.g., Atlantic cod *Gadus morhua*^23^, bighorn sheep *Ovis canadensis*^24^, and sifakas *Propithecus verrauxi*^25^) taxa, and there is some evidence that testis size influences offspring production as well (e.g., Soay sheep^21^, yellow-pine chipmunks *Taimias amoneus*^26^). Some male traits are influenced by both male-male competition and female choice (e.g., body size in carrion flies *Prochylzia xanthostma*^27^, body size in pond dragonflies *Libellua luctosa*^28^). Additionally, male morphology may be affected by evolutionary trade-offs between investment in traits associated with pre- and post-copulatory selection (e.g., weaponry and testes across cetacean species^29^, body mass and genital size across species of pinnipeds^30^, ornaments and testis volumes across primate species^12^, ornaments and sperm swimming speed in guppies^31^, hyoid volume and testis size in howler monkeys *Alouatta spp*.^32^).

Primates are a good taxonomic group in which to investigate how sexual selection pressures shape phenotypic variation. Sexual dimorphism in body and canine size^33–35^ and relative testis volume^6^ vary widely across species, suggesting variation in investment in both direct male-male competition and in sperm competition across the Order. The function of color ornaments also varies across species – some primarily function as “badges of status”^36–39^ and seem to have evolved under male-male competition, while others appear to be selected through both male-male competition and female choice (e.g., mandrills^40^, rhesus macaques^41–44^). However, the degree to which male-male competiton and female choice have influenced the evolution of primate morphological traits generally, or indeed whether primate morphological traits are evolving under any mechanism of sexual selection, remains largely unknown.

Rhesus macaques (*Macaca mulatta*) are a useful primate species in which to examine how sexual selection mechanisms influence male morphology because they appear to experience multiple sexual selection pressures. Rhesus macaques live in multi-male multi-female social groups and have a polygynandrous mating system^45^. Males acquire dominance through queueing rather than contest, such that the most dominant males are not always the strongest or highest quality in the group^46^. Relatedly, dominance rank is not usually a strong predictor of reproductive success among male rhesus macaques and reproductive skew is low in males as well^47,48^. Furthermore, there are multiple routes to reproductive success in rhesus males: some males engage in consortships while others employ alternative reproductive tactics, like sneaky matings, and both very passive and very aggressive males sire offspring^49–52^.

Rhesus macaque morphology most likely reflects the effect of a unique suite of sexual selection pressures. Rhesus macaques are moderately dimorphic in body mass (males are ~44% larger than females) and more strongly dimorphic in canine length (male canines are ~207% longer than female canines)^53^, reflecting investment in direct male-male competition. Male rhesus macaques also have large testis volumes for their body size, indicating a role for sperm competition, and they accumulate body fat prior to the mating season, enabling them to undertake costly mating strategies like consortships, as a form of endurance rivalry^6,54,55^. In additional to body fat, several other male traits show seasonal variation: males exhibit the largest testis volumes, highest androgen concentrations, and deepest facial coloration during the mating season^56–58^. Finally, male red facial coloration is attractive to females and influences reproductive success in high-ranking males^41–43^. To date however, whether most of these traits (with the exception of facial coloration^41^), are genetically inherited or influence reproductive success, and hence if they might be evolving under sexual selection – is unknown.

Here, we explored whether morphometric traits in male rhesus macaques are evolving under sexual selection. We quantified trait heritability (1), as variation in a trait must be heritable in order for the trait to respond to selection. We also investigated potential evolutionary and developmental trade-offs (2) between investment in different traits. We investigated the impact of seasonality (2a) on trait values, correlations between traits (2b) and correlations between dominance rank and trait values (2c). We predicted that: 2a) males captured later in the trapping season would be of greater body mass, higher in body fat, and exhibitlarger testis volumes, reflecting increasing investment in these traits leading up to the mating season; 2b) testis volume and measures of weaponry (body size and canine size) would be inversely correlated across males; and 2c) that dominance rank would not be correlated with trait values. Finally, we quantified selection on traits (3) by measuring relationships between trait values and reproductive success.

## Results

### Trait heritability

In our models using data from both parents, all measurements except testis volume were heritable (h^2^ ≥ 0.1^59^, Table 1a). DIC values for models including the animal (heritability) random effect were lower than DIC values for all models excluding the animal term, indicating that the addition of the heritability term produced a better fitting model (Table 1a,b), even though the testis volume heritability estimate did not meet our threshold to be considered heritable. Testis volume heritability estimates were extremely low (h^2^ < 0.1), and about half of the variance in testis volume could be explained by the date the animals were measured (see more details below). Date measured was not a significant contributor to variance in any of the other traits. Confidence intervals for the heritability and maternal effects were wide, so we interpret the HDPI values with caution, given the very low lower limits of these intervals. None of our sex-linked heritability models converged.

**Table 1:** **1a**. Results from heritability models for morphometric traits. HDPI refers to the highest density posterior interval for the estimate. Random effects with a posterior mode of 0.3 or higher and fixed effects with a pMCMC value of < 0.05 are highlighted in bold. **1b.** Results from models without the animal (heritability) random effect term. HDPI refers to the highest density posterior interval for the estimate. Random effects with a posterior mode of 0.3 or higher and fixed effects with a pMCMC value of < 0.05 are highlighted in bold.

1a	Crown-Rump Length (n = 107 males; n = 21 females)	Body Mass (n = 108 males; n = 21 females)	Upper Canine Length (n = 114 males; n = 21 females)	Testis Volume (n = 97)	Abdominal Skinfold Thickness (n = 108 males; n = 21 females)
Heritability (HDPI)	0.183 (0.051 – 0.565)	**0.396 (0.038 – 0.648)**	**0.513 (0.167 – 0.704)**	0.079 (0.015 – 0.384)	**0.502 (0.066 – 0.753)**
**Random effects**					
Maternal ID (HDPI)	0.283 (0.054 – 0.568)	0.101 (0.037 – 0.589)	0.133 (0.051 – 0.357)	0.073 (0.019 – 0.364)	0.105 (0.028 – 0.476)
Date Measured (HDPI)	0.112 (0.035 – 0.2)	0.128 (0.038 – 0.302)	0.077 (0.036 – 0.203)	**0.406 (0.200 – 0.651)**	0.082 (0.026 – 0.226)
**Fixed effects**					
Age (pMCMC)	0.370	0.057	0.635	0.298	**0.033**
Sex (pMCMC)	**<0.001**	**<0.001**	**<0.001**	n/a	0.454
DIC	176.921	158.824	117.158	173.292	222.911
**1b**	**Crown-Rump Length (n = 107 males; n = 21 females)**	**Body Mass (n = 108 males; n = 21 females)**	**Upper Canine Length (n = 114 males; n = 21 females)**	**Testis Volume (n = 97)**	**Abdominal Skinfold Thickness (n = 108 males; n = 21 females)**
**Random effects**					
Maternal ID (HDPI)	**0.503 (0.108 – 0.705)**	**0.628 (0.161 – 0.792)**	0.196 (0.085 – 0.507)	0.126 (0.022 – 0.453)	0.109 (0.039 – 0.603)
Date Measured (HDPI)	0.140 (0.044 – 0.301)	0.132 (0.045 – 0.316)	0.091 (0.040 – 0.221)	**0.489 (0.219 – 0.662)**	0.096 (0.021 – 0.234)
**Fixed effects**					
Age (pMCMC)	0.351	**0.034**	0.539	0.264	**0.023**
Sex (pMCMC)	**<0.001**	**<0.001**	**<0.001**	n/a	0.336
DIC	233.496	221.144	217.748	217.468	338.041

### Evolutionary and developmental trade-offs

#### Seasonality

**Figure 1:**
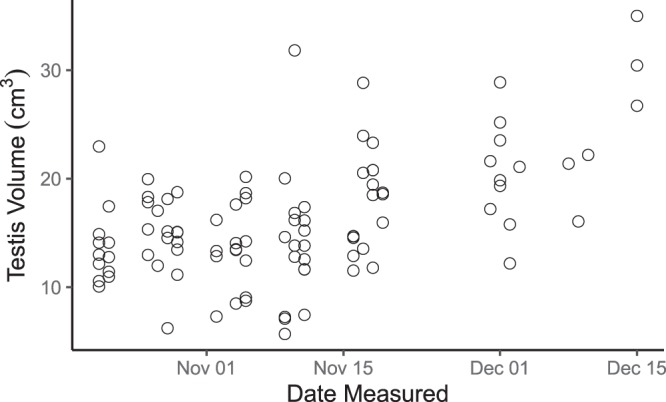
Seasonal increases in testis volume across the trapping season (n = 97). Data points represent measurements from individual males.

Date measured influenced three of our traits of interest: body mass, testis volume, and crown-rump length (Table 2). Males measured later in the trapping period had higher body masses, longer crown-rump lengths, and larger testis volumes (Fig. 1). Date measured did not influence measurements of canine length or abdominal skinfold thickness. These results contrast with those from our heritability models, where we treated date measured as a random, rather than a fixed effect. In the heritability models, date measured only contributed to a large proportion of the variation in testis volume.

**Table 2:** GLM results for effects of seasonality on morphometric traits. Statistically significant terms (p < 0.05) are shown in bold.

Variable	Estimate	Standard error	t-value	p-value
**Crown-Rump Length**				
Intercept	28391.600	11391.600	2.492	**0.014**
Age	0.042	0.068	0.613	0.541
Date Measured	0.040	0.016	2.488	**0.014**
**Body Mass**				
Intercept	−596.957	176.936	−3.374	**0.001**
Age	−0.082	0.045	−1.823	0.071
Date Measured	0.036	0.011	3.441	**<0.001**
**Upper Canine Length**				
Intercept	−934.241	1435.460	−0.651	0.517
Age	−0.204	0.088	−2.324	**0.022**
Date Measured	−0.013	0.021	−0.652	0.516
**Testis Volume**				
Intercept	−3368.727	561.243	−6.002	**<0.001**
Age	0.206	0.151	1.360	0.177
Date Measured	0.202	0.035	6.026	**<0.001**
**Abdominal Skinfold Thickness**				
Intercept	−131.997	317.812	−0.415	0.679
Age	−0.175	0.081	−2.168	**0.032**
Date Measured	0.008	0.019	0.441	0.660

#### Correlations between traits

In our dataset, body mass was significantly correlated with crown-rump length, testis volume and abdominal skinfold thickness (Table 3, Supplementary Fig. S1). Testis volume (either absolute or relative) was not correlated with either abdominal skinfold thickness or canine length (Table 3). Because relationships involving testis volume did not change after controlling for body mass, we used absolute testis volume in all subsequent analyses. Age was a significant term in all of the models, and was always negative.

**Table 3:** GLMs testing relationships between traits, controlling for age and date measured. Statistically significant terms (p < 0.05) are shown in bold. P-values were adjusted for multiple tests following the method of Benjamni and Hochberg^88^, which controls the false discovery rate.

Variable	Estimate	Standard error	t-value	p-value
**Body Mass (Cube Root)**				
Intercept	−23.472	9.887	−2.374	0.052
Crown-Rump Length	0.021	0.004	5.816	**<0.001**
Age	−0.007	0.002	−2.816	**0.027**
Date Measured	0.001	0.0006	2.484	**0.045**
**Body Mass**				
Intercept	−316.545	209.453	−1.511	0.238
Testis Volume	0.083	0.033	2.533	**0.045**
Age	−0.085	0.048	−1.762	0.186
Date Measured	0.019	0.013	1.560	0.238
**Body Mass (Cube Root)**				
Intercept	−38.104	13.05	−2.920	**0.023**
Canine Length	0.009	0.004	2.243	0.067
Age	−0.004	0.003	−1.189	0.380
Date Measured	0.002	0.0007	3.082	**0.017**
**Body Mass (Cube Root)**				
Intercept	−34.821	9.012	−3.863	**0.002**
Abdominal Skinfold Thickness	0.023	0.003	8.277	**<0.001**
Age	−0.002	0.002	−0.752	0.640
Date Measured	0.002	0.0005	4.096	**<0.001**
**Canine Length**				
Intercept	187.173	389.701	0.480	0.741
Absolute Testis Volume (Cube Root)	0.254	1.117	0.227	0.847
Age	−0.272	0.105	−2.592	**0.045**
Date Measured	−0.010	0.023	−0.425	0.741
**Canine Length**				
Intercept	89.403	373.069	0.240	0.847
Relative Testis Volume (Cube Root)	−1.226	2.530	−0.485	0.741
Age	−0.262	0.106	−2.471	**0.045**
Date Measured	−0.004	0.022	−0.178	0.859
**Abdominal Skinfold Thickness**				
Intercept	159.384	356.583	0.447	0.741
Absolute Testis Volume (Cube Root)	1.644	1.085	1.514	0.238
Age	−0.143	0.085	−1.680	0.205
Date Measured	−0.009	0.021	−0.434	0.741
**Abdominal Skinfold Thickness**				
Intercept	−239.697	338.016	−0.709	0.640
Relative Testis Volume (Cube Root)	−2.672	2.480	−1.077	0.433
Age	−0.104	0.087	−1.199	0.380
Date Measured	0.015	0.020	0.738	0.640

#### Correlations between dominance rank and trait values

There was no relationship between current ordinal dominance rank (low, medium, high) and any morphometric trait (ANOVA, p > 0.05, Supplementary Table S1).

### Selection on traits

We found no definitive evidence for directional, stabilizing, disruptive, or correlational selection on any of our focal morphometric traits (Tables 4–6a,b). Neither age nor date measured influenced relationships between trait values and reproductive success. Dominance rank (as assumed from dispersal rate) had a minor effect in several of our models, but the term was not statistically significant (0.05 < p < 0.10).

**Table 4:** Linear selection gradients (GLMs) for morphometric traits. Selection gradients are shown as the estimate +/− the standard error. Statistically significant terms (p < 0.05) are shown in bold. *β* refers to the linear selection gradient, t to the t-value, and p to the p-value.

	Crown-Rump Length	Body Mass	Upper Canine Length	Testis Volume	Abdominal Skinfold Thickness
Selection Gradient	$\beta$ = 0.127 $\pm$ 0.084 t = 1.525 p = 0.130	$\beta$ = 0.147 $\pm$ 0.085 t = 1.726 p = 0.087	$\beta$ = 0.055 $\pm$ 0.088 t = 0.622 p = 0.535	$\beta$ = 0.117 $\pm$ 0.099 t = 1.138 p = 0.257	$\beta$ = 0.052 $\pm$ 0.084 t = 0.627 p = 0.532
Age	t = 0.730 p = 0.468	t = 0.975 p = 0.332	t = 0.940 p = 0.349	t = 1.153 p = 0.252	t = 0.838 p = 0.430
Dominance Rank	t = −1.910 p = 0.059	t = −1.750 p = 0.083	t = −1.678 p = 0.097	t = −1.951 p = 0.054	t = −1.699 p = 0.092
Date Measured	t = −0.049 p = 0.961	t = −0.116 p = 0.908	t = −0.189 p = 0.850	t = −0.440 p = 0.661	t = 0.422 p = 0.673

**Table 5:** Quadratic selection gradients (GLMs) for morphometric traits. Selection gradients are shown as the estimate +/− the standard error. Statistically significant terms (p < 0.05) are shown in bold. $\gamma {ii}$ refers to the quadratic selection gradient, t to the t-value, and p to the p-value.

	Crown-Rump Length	Body Mass	Upper Canine Length	Testis Volume	Abdominal Skinfold Thickness
Selection Gradient (Quadratic Term)	$\gamma {ii}$ = −0.053 $\pm$ 0.052 t = −1.03 p = 0.305	$\gamma {ii}$ = −0.011 $\pm$ 0.055 t = −0.190 p = 0.850	$\gamma {ii}$ = −0.048 $\pm$ 0.054 t = −0.881 p = 0.381	$\gamma {ii}$ = 0.029 $\pm$ 0.057 t = 0.518 p = 0.606	$\gamma {ii}$ = −0.096 $\pm$ 0.050 t = −1.911 p = 0.059
Linear Term	t = 1.752 p = 0.083	t = 1.634 p = 0.105	t = 0.455 p = 0.650	t = 0.869 p = 0.387	t = 1.578 p = 0.117
Age	t = 0.753 p = 0.453	p = 0.326	t = 0.918 p = 0.361	t = 1.62 p = 0.248	t = 1.125 p = 0.263
Dominance Rank	t = −1.662 p = 0.100	t = −1.729 p = 0.087	t = −1.609 p = 0.111	t = −1.958 p = 0.053	p = 0.092
Date Measured	t = −0.007 p = 0.994	t = −0.128 p = 0.898	t = 0.113 p =0.910	t = −0.512 p = 0.610	t = 0.390 p = 0.697

**Table 6:** **6a.** Correlational selection gradients (GLMs) for morphometric traits and dominance rank.

6a	Crown-Rump Length and Dominance Rank	Body Mass and Dominance Rank		Upper Canine Length and Dominance Rank	Testis Volume and Dominance Rank	Abdominal Skinfold Thickness and Dominance Rank
Selection Gradient	*γij* = 2.696 ± 3.691 t = 0.730 p = 0.466	*γij* = 6.897 ± 4.639 t = 1.487 p = 0.140		*γij* = 2.973 ± 4.896 t = 0.607 p = 0.545	*γij* = 1.448 ± 5.802 t = 0.249 p = 0.803	*γij* = 4.997 ± 4.801 t = 1.041 p = 0.300
Age	t = 0.725 p = 0.449	t = 1.028 p = 0.306		t = 0.957 p = 0.341	t = 1.265 p = 0.209	t = 0.924 p = 0.358
Dominance Rank	t = −1.880 p = 0.063	t = −1.603 p = 0.112		t = −1.666 p = 0.099	t = −1.932 p = 0.056	t = −1.623 p = 0.108
Date Measured	t = 0.269 p = 0.788	t = −0.009 p = 0.993		t = −0.171 p = 0.865	t = 0.042 p = 0.966	t = 0.374 p = 0.709
**6b**	**Crown-Rump Length and Body Mass**	**Body Mass and Testis Volume**	**Body Mass and Upper Canine Length**	**Body Mass and Abdominal Skinfold** **Thickness**	**Canine Length and Testis Volume**	**Abdominal Skinfold Thickness and Testis Volume**
Selection Gradient	*γij* = −0.011 ± 0.081 t = −0.134 p = 0.894	*γij* = 0.106 ± 0.087 t = 1.226 p = 0.223	*γij* = −0.078 ± 0.089 t = −0.873 p = 0.385	*γij* = −0.038 ± 0.059 t = −0.647 p = 0.519	*γij* = −0.0005 ± 0.075 t = −0.006 p = 0.995	*γij* = 0.141 ± 0.106 t = 1.323 p = 0.189
Age	t = 0.723 p = 0.471	t = 1.256 p = 0.212	t = 0.876 p = 0.383	t = 0.847 p = 0.399	t = 1.573 p = 0.120	t = 1.319 p = 0.190
Dominance Rank	t = −1.769 p = 0.080	t = −1.961 p = 0.053	t = −1.607 p = 0.111	t = −1.842 p = 0.068	t = −1.969 p = 0.052	t = −1.930 p = 0.057
Date Measured	t = 0.403 p = 0.688	t = 0.068 p = 0.946	t = −0.112 p = 0.911	t = 0.511 p = 0.610	t = −0.122 p = 0.903	t = 0.288 p = 0.773

Selection gradients are shown as the estimate +/− the standard error. Statistically significant terms (p < 0.05) are shown in bold. *γіj* refers to the correlational selection gradient, t to the t-value, and p to the p-value. **6b.** Correlational selection gradients (GLMs) for combinations of morphometric traits. Selection gradients are shown as the estimate +/− the standard error. Statistically significant terms (p < 0.05) are shown in bold. *γіj* refers to the correlational selection gradient, t to the t-value, and p to the p-value.

## Discussion

We used a quantitative genetic approach to investigate the production and maintenance of variation in male rhesus macaque morphometric traits putatively associated with intrasexual competition. Our results suggest that male morphometric traits are heritable, but that variation in these traits does not predict reproductive success. We also found that male morphometrics were not influenced by dominance rank, and we found no evidence for trade-offs in investment between morphometric traits.

For several traits, either additive genetic variance (heritability) or maternal ID contributed to a moderate proportion of the phenotypic variation in the trait. Canine length, body mass, and abdominal skinfold thickness all had moderate heritability values (h^2^ > 0.3), though crown-rump length values were lower (h^2^ < 0.2). We found very low additive genetic variance in testis volume. It is possible that intraindividual seasonal increases in testis volume have confounded our results. However, low additive genetic variance may also be the result of strong selection on testis volume, as strong selection may erode additive genetic variation^60^. Additionally, all of our estimates for heritability and maternal effects have wide confidence intervals, ranging from very low (0.02) to quite high (0.75) in some cases. Future studies with larger samples might help to resolve these values with greater confidence. Overall, our results suggest that variation in most male morphometric traits has a genetic basis, and as such, that these traits can evolve under selection.

We found no evidence for trade-offs between investment in different morphometric traits. Body mass was positively correlated with crown-rump length, testis volume, and abdominal skinfold thickness. These correlations likely reflect allometric relationships, not investment in multiple reproductive strategies, and are consistent with previous work on male morphometric traits in rhesus macaques^61^. We found no correlation between canine length and testis volume, and we also found that jointly, these traits did not explain variation in reproductive success, suggesting that inter-individual variation in male morphology does not reflect relatively higher investment in either direct or sperm competition. These findings do not support our prediction that testis volume would be inversely correlated with variation in body size (mass and length) and canine size. This finding is consistent with inter-specific analyses that have shown that species with lower levels of direct male-male competition do not exhibit strong trade-offs between investment in weaponry and investment in testis volume^10^ but contrasts with intra-specific studies of other taxa, which have clearly demonstrated trade-offs between traits that are involved in pre- and post-copulatory competition^31,32,62^. This finding may also reflect different patterns of investment in canines and testes: once canines are formed, no additional energetic investment is required – in contrast, testes need to be maintained throughout adult life^12^. We would need many data points collected across the lifespan of individual males in order to test this idea. Furthermore, our prediction that current dominance rank was not correlated with morphometrics was supported, providing further evidence that competitive ability is not important for dominance acquisition in this species. Lastly, these analyses revealed an effect of age on variation in morphology. Age was always a negative term in the models, suggesting that older animals are smaller than younger ones. We cannot determine whether this reflects the aging process, cohort effects, or selective mortality of particular male phenotypes with our data. Additional work is necessary to address whether smaller males are more likely to survive to older ages.

Our prediction that body mass, testis volume, and fat mass would be higher in males captured later during the capture-release period was partially supported. We found that males captured later had higher testis volumes than those captured earlier, which confirms prior work demonstrating that rhesus males undergo dramatic increases in testis volume prior to the mating season, indicating strong investment in sperm competition^56^. We also found that males trapped later in the trapping season had higher body masses and longer body lengths, but not higher fat mass. This finding may either reflect seasonal increases in body size or an effect of body size on a male’s ability to be captured. In order to test for seasonal increases in body size, we would need to collect data on body size in the months leading up to the mating season. Since it is not possible to trap animals on Cayo Santiago during this period, one possible way of doing this would be to collect multiple body length measurements per male (e.g., one measurement per week in the three months leading up to the mating season) using photogrammetric methods (e.g., Breuer *et al*.^63^; Wright *et al*.^64^). Alternatively, this result may reflect the fact that larger males happen to be trapped later than smaller ones because they are harder to capture.

We did not find evidence of selection on any of our morphometric traits. Our results echo those from Atlantic cod^23^, bighorn sheep^24^, and sifakas^25^ but contrast with those from mandrills^22^, Soay sheep^21^, red deer^20^, kangaroos^19^, minnows^18^, and field crickets^16^, among others. Our findings provide additional evidence that even in sexually dimorphic species, larger or more highly-weaponized males do not always enjoy the highest reproductive success. Rather, among rhesus macaques, female preference (such as that based on facial coloration^41,43^) and male behavioural strategies^51,52^ are likely stronger predictors of male reproductive success.

Our results could be interpreted in multiple different ways. One interpretation is that the measured male morphometric traits are not under selection. Alternatively, the results are also largely consistent with previous evidence of flat fitness landscapes and multiple routes to male reproductive success in male rhesus macaques^51,52^. Under these scenarios, we are unlikely to find clear linear or quadratic relationships between specific traits and reproductive success, because there are multiple routes to equal levels of success. We found weak, but not statistically-significant, evidence that male dominance rank influences reproductive success independent of variation in male morphometric traits, consistent with previous analyses of reproductive skew^47^. Because rhesus males do not contest dominance, this result provides further evidence that direct male-male competition is not a strong selection pressure in this species. In general, our results confirm prior research indicating that variation in morphological traits associated with competition is not a strong predictor of reproductive success in this species^54,61^. Additional studies are necessary to determine whether male reproductive strategies or aggression levels are correlated with morphology.

Our findings illustrate that in species that are evolving under multiple sexual selection pressures, such as rhesus macaques, male traits like large body size, enhanced weaponry, and large testis volumes may not increase reproductive success through linear, quadratic, or correlational selection. Our results highlight the importance of understanding how interactions between sexual selection pressures, and between behavior and morphology, function to influence male reproductive success.

## Methods

### Field site and subjects

Cayo Santiago is a 15.2 hectare island located off the southeast coast of Puerto Rico. The Caribbean Primate Research Center (CPRC) manages the island and the population of free-ranging rhesus macaques that live there^65^. At the time this study was conducted, the island was inhabited by ~1,500 rhesus macaques divided into seven naturally-formed social groups, all of which descend from a founding population of 409 animals brought to the island from India in 1938^65^. Even though no outside animals have been introduced into the colony, the population is not inbred^66^. The CPRC monitors the population daily and maintains long-term (>75 years) behavioural and demographic databases including data on social group membership for all animals, plus a genetic parentage database for animals born after 1985^65,67,68^. Each year, before the onset of the mating season, a subset of the animals ranging on the island are captured for collection of blood samples and morphometric data, and then released. During the capture-release period, all one-year-old animals are captured, sampled for blood, assigned a unique ID, and tattoed, enabling researchers to easily identify individual animals.

### Morphometric data collection

We collected morphometric data from male and female rhesus macaques (n = 146) captured during the annual capture-release period (October 15, 2015 to December 15, 2015). Our sample is composed of all adult males (ages 6 and above) ranging on the island who were able to be captured (n = 125), but we also collected data on females closely related to the males we sampled (n = 21) for use in our heritability analyses. We collected a set of measurements on focal traits that could act as proxies for different types of male-male competition: crown-rump length (direct contest competition), body mass (direct contest competition and endurance rivalry), canine length (direct contest competition), testis dimensions (sperm competition), and upper abdominal skinfold thickness (endurance rivalry). We chose these traits based on prior studies of sexual selection in rhesus macaque males^54,61^. Animals were only captured and measured once during the capture-release period. All measurements were collected by one trained observer. Body weight was measured using a hanging scale and all other measurements were collected using either a tape measure or digital calipers (accurate to 0.01 mm). Weight (lbs) was converted to mass (kg) for all analyses. Testis volume was calculated from three dimensions: height (h), width (w), and depth (d) and modeled as an ellipsoid: $V=\frac{4}{3}\pi hwd$ ^69^. We calculated relative testis volume by dividing testis volume by body mass. We excluded one measurement of an extremely worn or broken canines, one crown-rump length value that was three standard deviations above the mean and likely an error, and measurements affected by pathological conditions (n = 12) from our analyses.

### Genetic parentage information

The CPRC maintains a pedigree database containing information on behavioural dams (available for all animals) as well as genetic parentage assignments for dams and sires (available for animals born after 1985). Genetic parentage assignments are made based on a panel of microsatellites^48^. We used the R package MasterBayes to prune the full Cayo Santiago pedigree so that it only included phenotyped animals and those individuals that provided connections between them^70^. Our pruned pedigree spanned ten generations and included 902 animals, with 885 maternities and 567 paternities (pedigree statistics were generated using the pedantics R package^71^).

We used average number of offspring produced per year as a proxy for male reproductive success. We then calculated relative annual reproductive success (each animal’s reproductive success divided by the average value across our entire sample) and used this measure in our selection gradient models^72^. We could not use lifetime reproductive success because the majority of our study animals had not yet reached reproductive senescence (>17 years of age^48^).

### Dominance rank

We quantified dominance rank two different ways: first, over the course of one year and second, as an average measure over the animal’s life to date (up to when they were captured for morphometric measurements). We used current dominance rank (available for a subset of 55 adult males) to determine whether current rank and male morphology were correlated. We determined the dominance rank of all subjects using pairwise win-loss information from agonistic encounters that were recorded during focal animal samples or during ad libitum observations collected as part of an on-going, unrelated, study. We calculated dominance rank amongst males living within the same social group group. In order to account for variable group sizes, we then calculated dominance rank as the percentage of male groupmates that a subject outranked. We then classed males as either high, mid or low ranking based on this scale, with high ranking animals being those that outranked between 80–100% of males in their group, and low-ranking animals being those that outranked fewer than 49% of males in their group. We chose to bin the ranks this way because the behavioral data used to calculate these ranks are fairly coarse, so these categories are likely to be more accurate than continuous ranks, which may contain errors in exact rank order. Furthermore, the correlation between average dispersal rate and continuous dominance ranks, while strong, is not perfect, so binning the ranks makes the measures more comparable. Finally, this method has been used in previous studies on this population^73,74^ so conducting our analyses this way makes our study more comparable with prior work.

In our selection gradient analyses (n = 108), we chose to use an average measure of dominance rank – average annual dispersal rate – because our measure of reproductive success was also averaged over the animal’s life (up until they were captured)^41^ and we did not have the behavioral data necessary to calculate dominance ranks for many of the males in our sample. Dispersal rate is a good proxy for dominance rank because rhesus males acquire dominance through queuing instead of contest, such that a male’s dominance rank can be predicted by group tenure length^46^. In our dataset, dominance rank and tenure length were strongly correlated – males with longer tenure lengths were higher-ranked (Pearson’s product moment correlation = −0.579, n = 81, p < 0.001).

### Statistical analyses

All statistical analyses were run in R version 3.5.2^75^. We considered p-values to be significant if alpha levels were below 0.05. P-values for generalized linear models (GLMs) were calculated based on a Student t distribution, p-values for ANOVA models were calculated using the F distribution.

#### Trait heritability

We used animal models to estimate narrow-sense heritability values (*h*^2^) for our morphometric traits. Animal models are univariate generalized mixed models that combine phenotypic and pedigree data to parse out the contributions of additive genetic and environmental factors to variation in a trait^76,77^. We implemented our models in the R package MCMCglmm^78,79^. We ran models on a combined sample of both males and females for body mass, crown-rump length, canine length, and upper abdominal skinfold thickness. We included six-year old males in our canine heritability analyses, as canine formation and eruption is generally complete by age six^80,81^, while all other analyses were run on males age seven and above because body growth is generally not complete until age seven. We ran models on mean-scaled measurements. In the pooled sex models, we controlled for age and sex (fixed effects), plus maternal ID, animal ID, and date measured (random effects). We also ran models for testis volume; these included maternal ID, animal ID, and date measured (random effects) and age (fixed effect). Lastly, we ran models for males using only paternal pedigree data to test for sex-linked inheritance, using the same model structure as listed above. We included maternal ID to account for non-genetic differences in maternal care and date measured to account for any changes in morphology over the course of the capture-release period. Animal ID is used to calculate the additive genetic variance.

We ran models on each trait for 2,550,000 iterations with a burn-in period of 50,000 iterations and a thinning interval of 1,000. Although we ran our analyses using a range of prior types and structures, we report values from models with inverse Wishart priors (V = 1, nu = 0.2). Priors with lower values of nu (e.g., nu = 0.002) did not mix well – the chains were autocorrelated – and confidence intervals for random effect terms were very wide. In order to verify that models met assumptions regarding autocorrelation and convergence, we inspected plots of the MCMC chain, ran Heidelberg stationarity tests, and ensured that autocorrelation between estimates was less than 0.1^82,83^. We calculated narrow-sense heritability values (h^2^) by dividing the proportion of variation due to additive genetic variance (V_A_; the posterior distribution of the animal effect) by the total phenotypic variance (V_P_; the summed posterior distribution of the maternal effect, date effect, and residual variance). We also ran a set of models without the animal ID term. We then compared DIC values from models with animal ID and those without – models with the lower DIC value were considered to be the best fit models.

#### Evolutionary and developmental trade-offs

First, we explored how seasonality may influence our morphometric traits of interest, as this has direct implications for our ability to detect trade-offs between traits. We investigated whether the date an animal was measured was related to variation in morphometrics using generalized linear models (GLMs). We set the trait as the response variable and age and date measured as fixed effects.

We then investigated whether males exhibited trade-off between traits associated with different mechanisms of competition using GLMs. We examined relationships between body mass and testis volume, body mass and abdominal skinfold thickness, abdominal skinfold thickness and testis volume (both relative and absolute), canine length and testis volume (both relative and absolute), and canine length and body mass, controlling for age and date measured.

We also explored whether current dominance rank (categorical: low, medium, high) was related to variation in male morphology (crown-rump length, body mass, canine length, testis volume, relative testis volume, and abdominal skinfold thickness) using ANOVA tests. We controlled for age, date measured and social group in our analyses.

#### Selection on traits

We assessed whether male trait variation predicted variation in reproductive success (measured as average annual offspring production) using selection gradient models^84,85^. We calculated linear selection gradients to estimate directional selection on single traits, quadratic selection gradients to estimate disruptive or stabilizing selection on single traits, and correlational selection gradients to determine if trait values in combination with average annual dominance rank influenced reproductive success. We ran linear selection gradients using mean-standardized trait values, quadratic selection gradients on squared mean-standardized trait values^86^, and correlational selection gradients using mean-standardized values of one trait multiplied by mean-standardized values of another trait^87^. We ran correlational models for trait values and average annual dominance rank, and for pairs of morphometric traits. We used the cube-root of body mass and testis volume in these correlational gradients so that both of our morphometric variables of interest were on the same scale. We controlled for age, dominance rank, and date measured (fixed effects) in our models. We square-root transformed age and average annual dominance rank so that models met assumptions (normally-distributed residuals), but did not transform reproductive success values^84^. We included all males age seven or above in our selection analyses (n = 108), regardless of whether or not they had produced an offspring.

### Ethical statement

This work was conducted in accordance with the Animal Behavior Society guidelines. All protocols were approved by the Institutional Animal Care and Use Committee of the University of Puerto Rico (protocol A150116) and the University Animal Welfare Committee of New York University (protocol 14-1439).

## Supplementary information

Supplementary information

## Data Availability

The datasets generated during and/or analyzed during the current study are available at 10.6084/m9.figshare.11343971.
