# Supplementary material for: Male morphological traits are heritable but do not predict reproductive success in a sexually-dimorphic primate
Source: Sci Rep. 2019 Dec 24;9:19794. doi: 10.1038/s41598-019-52633-4 (PMC6930303; doi:10.1038/s41598-019-52633-4)
Supplement: Supplementary file 1 — Supplementary information [file 41598_2019_52633_MOESM1_ESM.pdf]

Supplementary Figure S1. Relationships between body mass and testis volume (n = 97).

Data points represent measurements from individual males.

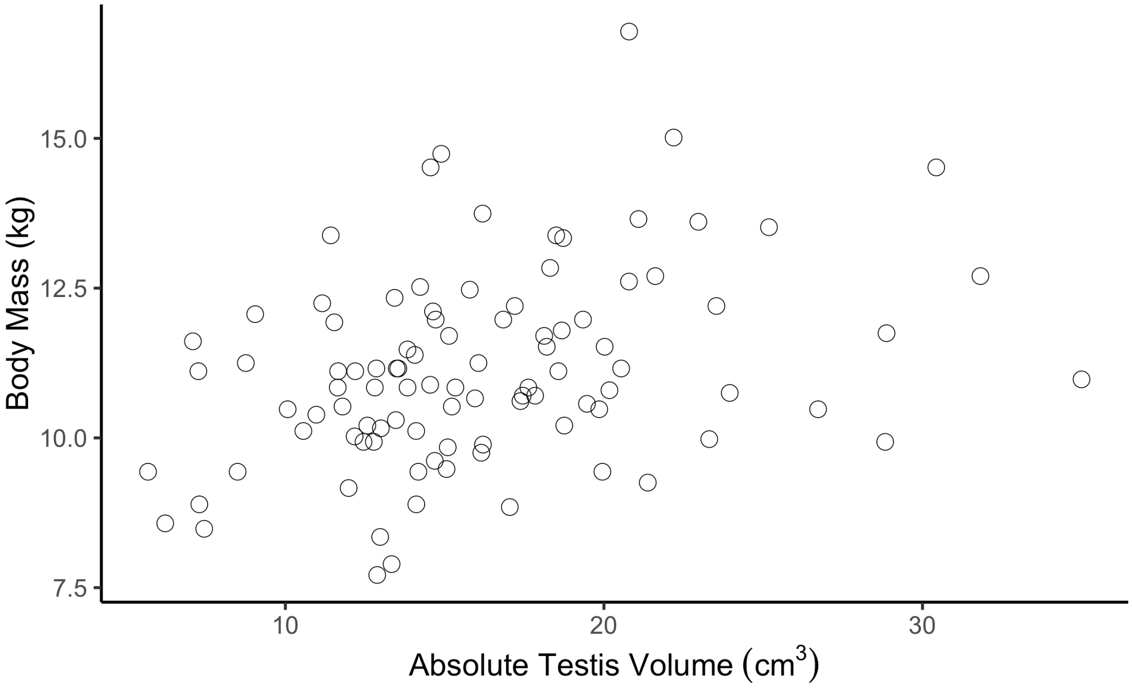

Supplementary Table S1. ANOVA results for relationships between dominance rank and morphometric traits

Variable	df	SS	mean square	F-value	Pr(>F)
Crown-Rump Length					
Ordinal Rank	2	7.792	3.896	0.595	0.556
Date Measured	1	13.171	13.171	2.013	0.163
Age	1	8.493	8.493	1.298	0.261
Group	5	45.727	9.145	1.398	0.244
Residuals	44	287.917	6.543		
Body Mass					
Ordinal Rank	2	13.287	6.643	0.527	0.594
Date Measured	1	103.532	103.532	8.219	**0.006**
Age	1	103.101	103.101	8.185	0.006
Group	5	30.634	6.127	0.486	0.785
Residuals	45	566.871	12.597		
Upper Canine Length					
Ordinal Rank	2	0.902	0.451	0.064	0.938
Date Measured	1	17.459	17.459	2.490	0.122
Age	1	68.595	68.595	9.785	**0.003**
Group	5	12.169	2.434	0.347	0.881
Residuals	38	266.401	7.011		
Testis Volume					
Ordinal Rank	2	35.350	17.675	0.599	0.554
Date Measured	1	694.972	694.972	23.562	**< 0.001**
Age	1	24.918	24.918	0.845	0.363
Group	5	56.096	11.219	0.380	0.860
Residuals	45	1327.309	29.496		
Abdominal Skinfold Thickness					
Ordinal Rank	2	8.020	4.010	0.438	0.648
Date Measured	1	7.105	7.105	0.776	0.383
Age	1	40.016	40.016	4.370	**0.042**
Group	5	34.709	6.942	0.758	0.585
Residuals	45	412.077	9.157		
